# Reduced Gut Bacterial Diversity in Early Life Predicts Feeding Intolerance in Preterm Neonates

**DOI:** 10.3390/tropicalmed9080174

**Published:** 2024-08-06

**Authors:** Maria Di Chiara, Alessandro Lazzaro, Daniela Scribano, Maria Trancassini, Valeria Pietropaolo, Michele Sonnessa, Chiara De Luca, Rita Prota, Elisa Onestà, Gianluigi Laccetta, Gianluca Terrin

**Affiliations:** 1 Department of Maternal Infantile and Urological Sciences, Sapienza University of Rome, Viale del Policlinico 155, 00161 Rome, Italygianluigi.laccetta@uniroma1.it (G.L.); 2 Department of Public Health and Infectious Diseases, Sapienza University of Rome, Viale del Policlinico 155, 00161 Rome, Italy; alessandro.lazzaro@uniroma1.it (A.L.); daniela.scribano@uniroma1.it (D.S.); maria.trancassini@uniroma1.it (M.T.);; 3 Bio-Fab Research Srl, Via Mario Beltrami, 5, 00135 Rome, Italy; michelesonnessa@biofabresearch.it

**Keywords:** microbiota, preterm neonate, feeding intolerance

## Abstract

Microbiota plays a crucial role in intestinal maturation in preterm newborns. The clinical manifestation of the immaturity of the gastro-intestinal tract is called feeding intolerance (FI). This condition may resolve spontaneously or dramatically evolve into necrotizing enterocolitis. One of the most challenging tasks for the neonatologist is to identify those neonates that will develop the disease early in order to adequately provide nutrition to these patients, from the very first hours of life. A close interplay between the maturity of the gastro-intestinal tract and gut microbiota has been described; however, in preterm neonates, this relationship is still undefined. We analyzed the bacterial composition of stool samples, collected early in life, from 30 preterm newborns classified as intolerant or tolerant according to the degree of readiness of the gastro-intestinal tract to receive enteral nutrition. The Pielou evenness index was significantly increased in intolerant compared with tolerant newborns. Data corrected for confounding variables confirmed that the occurrence of gut maturation was independently influenced by Pielou evenness at birth. A lower bacterial diversity very early in life is associated with improved feeding tolerance in preterm newborns. The abundance analysis showed that neonates not ready to receive enteral nutrition for feeding intolerance show, after birth, an increased abundance of *Proteobacteria*, *Lachnospiracae*, *Enterobacter* and *Acinetobacter*. We can argue that those are the taxa that prevent the establishment of pioneer bacteria. A lower alpha-diversity, in the first days of life, may facilitate the seeding of beneficial pioneer bacteria that, in turn, drive healthy microbial colonization during neonatal life.

## 1. Introduction

Preterm neonates suffer from immaturity of the gastro-intestinal tract that prevents most of them from receiving an adequate nutritional intake via the enteral route from the first days of life. A close interplay between the maturity of the gastro-intestinal tract and gut microbiota has been described [1,2,3,4]. The human gut microbiota is dynamic, shaped by multiple factors and has been shown to play an important role in human health. Early life is a pivotal time for the development of the gut microbial community and the host immune system, concomitant with the maturation of the gastro-intestinal system [5,6]. 

Moreover, compared to full-term infants, hospitalized preterm babies face unique challenges due to prematurity and adverse environmental and host conditions before and after birth. As a result, their gut microbiota is unique and shows substantial inter-individual variation [7]. The microbiota is known to be essential for the development of human life and is not only greatly involved in various indispensable physiological activities, such as metabolic processes and immune responses, but also closely associated with the occurrence of multiple diseases. Anomalous intestinal microbiota is supposedly associated with intestinal dysfunction, but the details of this relationship remain poorly understood, particularly in preterm newborns [8]. The acquisition of pioneer bacteria and their initial colonization are affected by a number of factors, including exposure to antibiotics and environmental conditions. Bacterial taxa that initially colonize the gut of newborns gradually make way for the colonization of further bacteria. Subsequent diversification and development of the microbiota continue dynamically until it reaches maturity. An important window of opportunity to affect the structure of developing microflora is likely to coincide with the first days of life [9]. Given that the postnatal development and assembly of the gut microbial community is associated with health benefits, it would be helpful if some microbial markers of normal and healthy progression of gut microbiota could be identified. Several studies have explored the microbial alterations in preterm infants with FI, revealing changes in microbial distribution and an increased relative abundance of some bacterial taxa [10,11,12,13]. However, the progression of gut colonization in preterm newborns, according to the degree of intestinal maturation, is still largely uninvestigated. In addition, due to variations in study populations, sampling time points and sampling frequency, definitive conclusions have not yet been drawn. Thus, there is indeed a lack of knowledge about the dynamic process of bacterial colonization of the intestinal tract and its relationship with intestinal maturity and feeding tolerance. Moreover, a fundamental issue hampering progress is that it is still unclear if the microbiota can influence the maturation of the gastro-intestinal tract.

The clinical manifestation of gastro-intestinal immaturity, in neonates, is called feeding intolerance (FI) and usually refers to a combination of clinical signs suggesting an inability by the subject to tolerate EN [14,15]. This condition may either resolve spontaneously, within the first weeks of life, or dramatically evolve into necrotizing enterocolitis (NEC). NEC is a major gastro-intestinal emergency in preterm newborns, characterized by a high risk of mortality and neurodevelopmental delay in survivors [16]. One of the most challenging tasks for the neonatologist is identifying preterm infants who will safely receive enteral nutrition (EN). The dilemma relies, indeed, on the fact that, on one hand, providing enteral feeds to a neonate with intestinal immaturity could increase the risk of NEC, but, on the other hand, withholding enteral feeds in a neonate can result in starvation drawbacks, such as increased risk of infections.

Starting from these considerations, we conducted a prospective study in a cohort of very preterm infants aiming to investigate the relationship between intestinal microflora composition in early life and the development of feeding intolerance in preterm babies. Specifically, the major goal of this study was to verify the relationship between the alpha-diversity index and feeding tolerance in this specific population.

## 2. Methods

### 2.1. Population and Sampling

This is an observational prospective study including inborn preterm neonates with gestational age (GA) less than 37 weeks, admitted to the Neonatal Intensive Care Unit (NICU) of “Umberto I” Hospital, Sapienza University of Rome. Newborns with at least one of the following conditions were excluded: (1) Apgar score < 5 at 5 min; (2) critical clinical conditions (pH < 6.8 on cord blood, or hypoxia with persistent bradycardia for at least 1 h); (3) incomplete clinical data; (4) maternal history of immunologic, inflammatory, or infectious diseases; (5) surgery; (6) major congenital malformations or inborn errors of metabolism; (7) neonatal congenital infections.

FI was diagnosed if any of the following symptoms occurred: (1) frequent vomiting (>3 times/day) or ‘‘coffee ground’’ vomiting; (2) unchanged or reduced feeding volume (lasting longer than three days); (3) feeding volume < 60 mL/kg/day at the end of the second week of life; or (4) fasting for more than two days because of the presence of mild signs [17]. NEC was diagnosed based on the Vermont Oxford Network criteria and staged according to Bell’s modified staging criteria [18].

Eligibility and enrolment were performed by neonatologists in charge, unaware of the study aims, on the basis of predefined criteria. Investigators not involved in the eligibility and enrolment phases prospectively recorded prenatal, perinatal and postnatal data (including modalities of EN administration) using a structured data form, from birth to discharge, transfer to another hospital, or death. Classification of neonates into tolerant and intolerant newborns and the diagnosis of NEC performed by neonatologists in charge were revised by two independent researchers. Definitive classification was confirmed after an agreement between three researchers (G.T., M.Di.C., R.P.).

Among the eligible subjects, who were prospectively enrolled, we selected (intolerant) newborns with signs and symptoms of FI as cases. In addition, those newborns without signs and symptoms of FI were classified as potential controls (tolerant). Each intolerant newborn was matched with one control according to their GA at birth. We planned collection of at least 0.5 g of formed stool in all eligible newborns as soon as possible after birth. All fecal samples were placed into sterile and codified tubes and stored at −80 °C. We selected fecal samples of 15 consecutive cases (intolerant) and 15 controls (tolerant). The main outcome was the variability in the alpha-diversity index between intolerant and tolerant neonates.

### 2.2. Feeding Protocol

Breast milk and preterm formula represented the two available options for enteral nutrition. Enteral feeding was started, with a minimal enteral feeding (MEF) (10–20 mL/kg/day divided into four to eight feeds) after 72 h of life, in case of stable clinical conditions. Our protocol recommends increasing feeds by 15–30 mL/kg/day every 24 h in the absence of FI. Donor human milk was not available in our NICU, during the study period. All subjects were evaluated daily. In the presence of signs and symptoms suggestive of NEC, enteral nutrition was suspended. PN was administered in all subjects through a central vascular access to maintain adequate fluid, electrolyte and nutrient intake until full enteral feeding (120 kcal/kg/day) was reached. Total amount of enteral and parenteral fluids was started at 70–100 mL/kg/day and advanced by increments of 20 mL/kg/day until 150–180 mL/kg/day. Probiotics were not routinely used, in our NICU, during the study period.

### 2.3. Ethics

This study was conducted in accordance with World Medical Association Declaration of Helsinki for medical research involving human subjects and after approval by the Ethics Committee of “Umberto I” University Hospital of Rome (number 5089). We collected anonymized data after written informed consent was obtained from the parents of each enrolled infant.

### 2.4. Metagenomic DNA Extraction, Illumina MiSeq Sequencing

DNA quantity and quality were evaluated by a NanoDrop spectrophotometer (Thermo Fisher Scientific, Waltham, MA, USA). DNA integrity was checked through 0.8% agarose gel electrophoresis in TAE buffer. 16S library construction and metagenomic sequencing were performed using Illumina MiSeq platform and 2 × 300 paired ends read strategy at Bio-Fab Research s.r.l. (Rome, Italy) (https://www.biofabresearch.com; accessed on 29 July 2024). Briefly, the hypervariable V3–V4 regions of bacterial 16S rRNA genes were amplified according to Klindworth et al., 2013. First, PCR reactions were carried out by producing a reaction mixture containing final 1× PCRBIO HiFi Buffer (PCR BIOSYSTEMS, USA) composed of 1 mM dNTPs and 3 mM MgCl_2_, 0.5 units of PCRBIO HiFi Polymerase (PCR BIOSYSTEMS, USA), 0.2 µM of each primer and 5 μL of DNA (10 ng/μL). Cycling conditions followed an initial denaturation at 95 °C for 3 min, 25 cycles of 95 °C (30 s), 55 °C (30 s), 72 °C (30 s), final extension at 72 °C for 5 min, and hold at 4 °C. After amplification step, a purification of amplicons was made through magnetic beads system. Indexing was then carried out by Nextera XT Index Kit, and PCR was performed with following conditions: denaturation at 95 °C for 3 min, 8 cycles of 95 °C for 30 s, 55 °C for 30 s, 72 °C for 30 s, final extension at 72 °C for 5 min and hold at 4 °C. After a further clean-up step, libraries were normalized and pooled, and sequencing was performed on Illumina MiSeq using a 2 × 300 flow cell V3 chemistry.

### 2.5. Statistical Analysis

Statistical analysis was performed using Statistical Package for Social Sciences Software for Microsoft Windows (SPSS Inc., Chicago, IL, USA), version 27.0 and Excel (version 16.48). We checked for normality using the Shapiro–Wilk test. The mean and standard deviation, or median and interquartile range, summarized continuous variables. We used chi-squared test for categorical variables, and Mann–Whitney U test for unpaired variables. The level of significance for all statistical tests was 2-sided (*p* < 0.05). After checking for assumptions, a binary regression analysis with a stepwise method was used to study the possible influence of confounding variables (birth weight < 1500 g, MEF, pH on cord blood, Pielou-evenness) on the occurrence of FI.

16S rRNA gene sequencing was performed to analyze the gut microbial diversity and composition of the infants. Alpha-diversity measures were estimated by i. Chao 1 index, ii. Shannon index, and iii. Simpson index, which identify, within individuals, taxa richness and evenness.

Standard nonparametric tests applied to relative abundance data lead to highly inflated FDRs (false-discovery rates). Thus, to perform the differential abundance analysis, we adopted the pipeline proposed by the Analysis of Compositions of Microbiomes with Bias Correction (ANCOM-BC). Based on Aitchison’s methodology, ANCOM-BC uses relative abundances to infer the absolute abundances and provides good performances controlling FDR while maintaining high power [19]. We adopted ANCOM-BC to identify differentially abundant taxa, to control the FDR for multiple testing and to provide 95% simultaneous confidence intervals for the mean differential abundance (DA) analysis of each taxon in the two experimental groups. These confidence intervals were adjusted for multiplicity using Bonferroni method. First, to focus on more abundant features, we filtered out bacterial taxa which were abundant in less than 20% of the samples. There are instances where some taxa are systematically absent in an ecosystem. For example, there may be taxa present among intolerant neonates that might be absent in tolerant neonates. If a taxon is considered to be a structural zero in an experimental group, then, for that specific ecosystem, the taxon is not used in further analysis. If a taxon is present in both intolerant and tolerant neonates, its abundance is compared between the two groups. ANCOM-BC analysis was performed using RStudio (Version 2022.12.0+353 Copyright © 2022 by Posit Software, PBC) and Microsoft Excel for Mac (Version 16.48). Other statistical analyses not involving microbiome 16S rRNA sequencing data were performed using the ‘‘stats’’ package in R (v.3.5.1).

Statistician was blinded to the study aims. On the basis of our preliminary results, we estimated a need for 15 participants in each group to obtain a power of 85% (type error = 0.05, drop out 10%, difference between cases and controls of 14 cent for at least one index of alpha-diversity).

## 3. Results

### 3.1. The Pielou Evenness Index between Tolerant and Intolerant Neonates

We included 15 intolerant neonates in this study, and, based on pairing criteria, we selected 15 tolerant neonates as controls. Among the 15 intolerant newborns, 1 was excluded because of congenital infection; similarly, 2 controls were excluded because of transfer to other hospitals. Thus, we analyzed the fecal samples of 14 neonates with the occurrence of FI (intolerant) and 13 neonates without FI (tolerant) (Table 1).

The main clinical characteristics of participating cases and controls are summarized in Table 1. Baseline clinical characteristics were similar between cases and controls. However, full enteral feeding (FEF) after 14 days of life was significantly more prevalent in intolerant neonates than controls (42.9% vs. 7.7%, *p* = 0.048). We found five neonates with an NEC diagnosis, with a mean GA of 30.6 ± 2.5 weeks and mean birth weight of 1408 ± 306.3 g.

**Figure 1:**
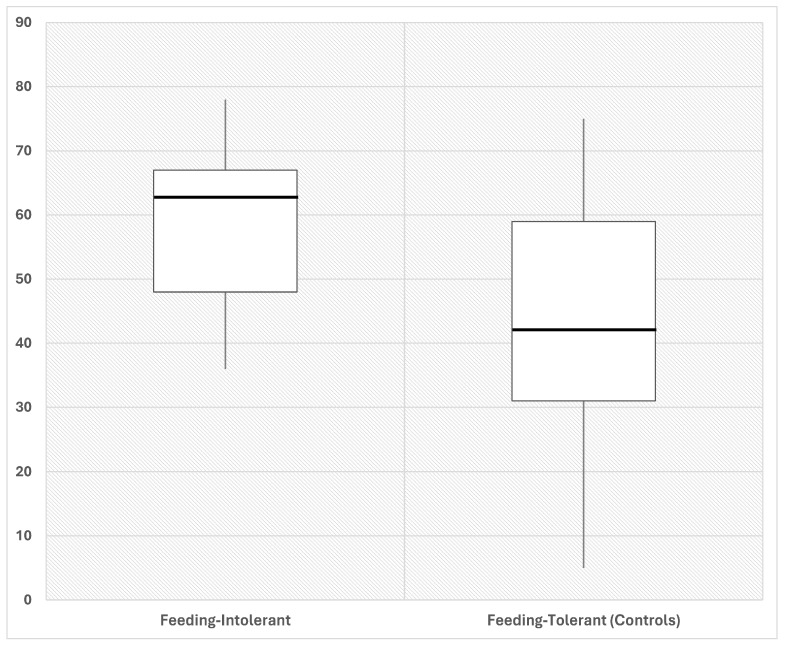
Representative box plot showing Pielou evenness in fecal samples of preterm neonates that developed FI (intolerant, *n* = 14) and controls without FI (tolerant, *n =* 13). Neonates that develop FI (intolerant) are characterized by increased Pielou evenness as compared to control neonates (tolerant), at early time post birth. Fecal samples were collected from each neonate as soon as possible after birth. Numbers indicate the measure of each index. *p* < 0.01, by Mann–Whitney test, between groups. Data expressed in %.

We assessed alpha diversity between the two groups using four indices. The Pielou evenness index was significantly (*p* < 0.05) higher in intolerant compared with tolerant neonates (Figure 1).

Conversely, we observed no differences in Group Faith (2.34 ± 2.1 vs. 2.57 ± 2.01, *p* = 0.78), Observed features (31.36 ± 28.7 vs. 34.84 ± 34.6, *p* = 0.77), and Shannon diversity (2.44 ± 0.79 vs. 1.98 ± 1.16, *p* = 0.24) indices. The linear regression analysis revealed that the Pielou evenness was significantly (*p* = 0.047) related to the occurrence of FI, even when corrected for confounding variables that have been reported to affect the risk of FI in preterm neonates (Table 2).

### 3.2. Relative Abundance of Taxonomic Features of Gut Microbiota between Tolerant and Intolerant Neonates

**Figure 2:**
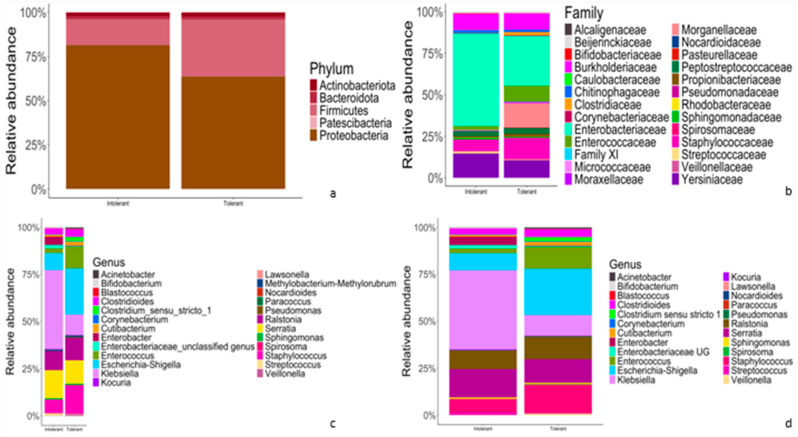
Comparison of relative abundances of taxonomic features of gut microflora between intolerant and tolerant neonates. (**a**–**d**) Representative bar plots showing the relative abundances of taxonomic features at phylum (**a**), family (**b**), genus (**c**) and species (**d**) rank, respectively. The left bar of each chart (**a**–**d**) represents intolerant newborns, whereas the right bar (**a**–**d**) accounts for tolerant neonates.

The results of classification and annotation at the phylum, family, genus and species levels of all samples are shown in Figure 2.

At the phylum level (Figure 2a), in both intolerant and tolerant neonates, the predominant phyla were *Proteobacteria* (81.4% in intolerant and 63.7% in tolerant neonates), followed by *Firmicutes* (14.7% in intolerant and 32.3% in tolerant). These two dominant phyla accounted for more than 95% of the bacteria in both groups (Figure 2a). At the family level, *Enterobacteriaceae* accounted for the highest proportion in both intolerant and tolerant newborns, followed by *Yersinaceae* and *Burkholderiaceae* in intolerant neonates and *Staphylococcacae* and *Burkholderiaceae* in tolerant patients (Figure 2b).

At the genus level, in intolerant neonates, the predominant genera were Klebsiella and Serratia, which accounted for more than 50% of bacteria (Figure 2c). In neonates with feeding tolerance, we observed *Escherichia-Shigella, Staphylococcus, Serratia, Ralstonia,* and *Enterococcus* as predominant genera (Figure 2c). These six genera accounted for more than 75% of the bacteria in controls, as shown in Figure 2c. Finally, we observed overlapping species abundances in both tolerant and intolerant neonates, characterized by unknown species (Figure 2d).

**Figure 3:**
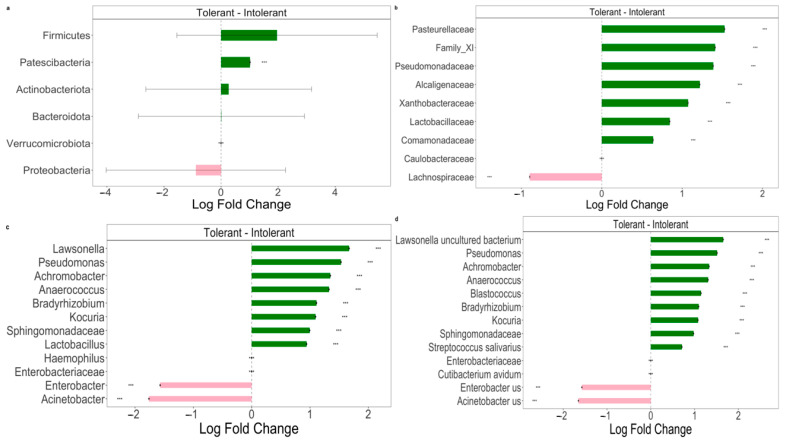
The Differential abundance (DA) analysis of gut microbiome between intolerant and tolerant neonates by taxonomic rank. (**a**) Pairwise differential abundance analyses at phylum rank. (**b**) Pairwise differential abundance analyses at family rank. (**c**) Pairwise differential abundance analyses at genus rank. (**d**) Pairwise differential abundance analyses at species rank. Data are represented by effect size (log fold change) and 95% confidence interval bars (two-sided; Bonferroni-adjusted) derived from the ANCOM-BC model. All effect sizes with adjusted *p* < 0.05 are indicated (*** significant at 0.1% level of significance). Diamonds on top of some bars indicate structural zeros. Pink bars, negative log fold change; green bars, positive log fold change.

Pairwise comparisons using ANCOM-BC analysis were performed to explore the relative abundance of taxa. The results are characterized by significant differences between intolerant and tolerant neonates’ specimens, as shown in Figure 3.

At the phylum level (Figure 3a), we observed a statistically significant difference in *Patescibacteria* phylum between intolerant and tolerant neonates. In particular, bacteria belonging to this phylum were significantly (*p* < 0.01) decreased in intolerant neonates compared with tolerant newborns (Figure 3a). Moreover, we observed a lower abundance of *Firmicutes* in intolerant with respect to tolerant neonates; vice versa, intolerant patients showed higher levels of *Proteobacteria* in comparison with tolerant neonates (Figure 3a). At the family level, we observed that the only *Lachnospiraceae* group was significantly (*p* < 0.01) higher in intolerant with respect to tolerant neonates (Figure 3b). At both the genus and species levels (Figure 3c,d), we observed that there were two genera and species groups (*Enterobacter* and *Acinetobacter*) that were significantly (*p* < 0.01) more represented in intolerant newborns, as compared to tolerant neonates.

## 4. Discussion

Preterm newborns with FI displayed a peculiar alpha diversity of gastro-intestinal tract microbiota resulting in higher Pielou evenness. Those babies showed an increased relative fecal abundance of *Protebacteria, Lachnospiracae, Enterobacter and Acinetobacter* in early life. This microbial composition is associated with an impairment in gut maturation.

Previous studies investigating the relationship between gut microbiota and gastro-intestinal maturation, in preterm neonates, mainly focused on the pathogenesis of NEC; however, none of these studies clearly addressed the role of microflora in either intestinal maturation or potential NEC onset and development [20,21,22]. A recent metanalysis including 14 studies reported that fecal microbiota from preterm infants with NEC had increased relative abundance of *Proteobacteria* and decreased relative abundances of *Firmicutes* and *Bacteroidetes* prior to NEC onset [23]. Metanalysis showed that alpha- or beta-diversity indices in preterm infants with NEC were not consistently different from controls [23]. However, in all previous studies, the gut microbiota was evaluated during the days immediately before the onset of NEC disease, when intestinal inflammation had already occurred; thus, the modifications in the intestinal environment due to inflammation were already in place, and this could have influenced the composition of microflora.

Few studies evaluated the intestinal microflora composition in children with FI, prior to the evolution in NEC disease [10,13,24]. Yuan et al. conducted a study to detect the features of the gut microbial community for feeding-intolerant preterm neonates [13]. They found a significantly increased diversity of intestinal microbiota according with the newborns’ days of life; however, no difference in terms of alpha diversity was found, early in life, before the onset of FI. Regarding the composition analysis, our results showed a low relative abundance of *Firmicutes* and a significantly increased relative abundance of *Proteobacteria* in feeding-intolerant preterm infants. However, the reported findings were not adjusted for confounding variables that may have affected gut colonization; moreover, there is a dearth of adequate analysis of the power of the study regarding alpha diversity.

More recently, two further studies aimed to evaluate differences in gut microflora, in preterm neonates, according with the occurrence of FI [10,24]. Similar to our study design, Liu et al. explored the relationships between the gut microbiota and FI in highly unstable preterm infants [10]. This case–control study analyzed differences in the composition of gut microbiota between preterm infants who developed FI and feeding-tolerant newborns from the control group. The alpha-diversity indices varied over time in both groups, showing a similar trend. Liu et al. found no difference in terms of alpha diversity between cases and controls, at 2 and 4 weeks of life. Hu et al. reported a lower alpha diversity in neonates with FI at 7 days of life, as compared with newborns showing tolerance to enteral nutrition. However, all these studies presented some significant limitations. The authors did not make a comparison in terms of alpha diversity between neonates with FI and those without this condition at similar time frames [10,13]. In both studies, fecal sampling was performed after 7 days of life, when the modification of gastro-intestinal flora leading to FI would have already occurred. Nevertheless, the authors did not perform multivariate analysis for the correction of confounding variables, and the sample power of the studies was not estimated [10,13]. In addition, the Pielou evenness was not evaluated.

Many studies reported the composition of the gastro-intestinal tract microbiota; however, none of them identified an accurate and specific profile of the gut taxa in neonates with intestinal immaturity [11,20,21,25,26,27,28]. Given the high heterogeneity of intestinal microbiota, and the struggle in correcting the results for confounding variables, we believe that this is not the “core” on which to focus. We concentrated our study on the role of microflora in the intestinal maturation of preterm newborns by collecting fecal samples within 24-48 hours of life, prior to the occurrence of intestinal dysfunction, potentially leading to necrosis. Our results suggest a crucial role for Pielou evenness in identifying newborns with a later onset of FI. The Pielou evenness is a measure of relative evenness of species’ richness [29]. The evenness of microflora describes relative differences in the abundance of various species in the community [30]. Our findings apparently disagree with previous research in the field, which described a lower diversity of intestinal microflora in neonates with the occurrence of FI. However, many observations may support our results. Theoretically, in neonates, a good and healthy microbiota would be a microbial consortium that leads to an improved neonatal outcome [31]. The gastro-intestinal tract of VLBW infants per se is not a “normal” natural habitat. It is indeed an unexplored habitat in preterm neonates who are in a fragile condition and not in a physiological environment [21,26,32]. Studies of malnourished infants have shown that maturation of the gut microbiota does not occur in a similar manner to healthy infants, even after dietary intervention, and it has been proposed that an ‘undernourished’ microbiome in infancy can perpetuate growth impairments later in life [33].

The intestinal microbiota refers to the collection of various microorganisms that inhabit the human intestine, and bacteria are the major members of the intestinal microbiota [34]. These pathogenic bacteria can promote the development of gastro-intestinal diseases by secreting harmful metabolites and toxins, disrupting the intestinal mucosal barrier, inhibiting immune cell function and the seeding of other bacteria with a crucial role in gastro-intestinal development [35,36].

There are conflicting reports in the literature regarding the ontogenesis of such beneficial microbiota composition and the microflora factors that shape gut maturation, especially among preterm infants [32].

With regard to microbiome ontology, interestingly, it has been hypothesized that the gut microflora ontogenesis could be influenced by some pioneering bacteria that prime and drive the colonization [25,37,38,39]. The same might be true for the gastro-intestinal tract; even in presence of a high alpha diversity, the absence of pioneer bacteria early in life may be associated with the lack of a healthy colonization in those newborns that will develop FI. A delay in the rate of assembly of intestinal bacteria and maturation of the gut bacterial population structure punctuated by abrupt shifts in microbial composition, potentially pathogenic microorganisms, have been reported in premature infants [25]. In addition, the first days after birth witness the competition represented by the microbial colonization of the gut by the first taxa, which gives birth to the very first preterm intestinal microbiota [40]. When a bacterial colony grows and reaches a certain size, it can divide and give rise to daughter colonies nearby; these daughter colonies are often referred to as sibling colonies because they originate from the same parent colony and share a genetic lineage [41,42]. Evidence has been provided about the competition between sibling colonies at the lumen–mucosa interface; in particular, intestinal bacteria can stimulate intestinal cells to release antibacterial peptides into the glycocalyx and lumen, which help to regulate the composition of colonizing intestinal bacteria [39,41,42]. Hence, we can speculate that the condition of intestinal immaturity may have an effect on the count of individual bacterial species, resulting in higher evenness in preterm infants at high risk, without affecting bacterial richness. Based on these observations, our results bring enough evidence to support the hypothesis that the intestinal colonization by beneficial pioneering bacteria might be crucial to provide pivotal ecological positive influences toward a proper intestinal maturation; on the contrary, even in the presence of a high alpha-diversity richness, the pathological progression towards food intolerance and NEC could be due to the lack of establishment of pioneering bacterial colonia overcoming other minor taxa (less represented within the microbial community at lower relative abundances, as compared to the pioneer bacteria). It is plausible that neonates with FI show increased intestinal microflora evenness that does not enable the growth that may help seeding of the infant gut microbiota. The high evenness shown by preterm neonates with FI means that microbial communities are pretty balanced between each other, without any competitor overcoming others, not allowing the establishment of pioneering bacterial colonia into the intestinal tract. In particular, the increased abundance of *Proteobacteria* in intolerant rather than tolerant newborns may not allow for the rise of some pioneer bacteria, leading to the establishment of a healthy microbiota. Conversely, a higher abundance of *Patescibacteria* and *Firmicutes* in tolerant neonates might allow for the growth of other taxa, such as *Lactobacillus*, that may represent those priming bacteria. The large number of randomized clinical trials on the administration of lactobacillus as a probiotic may find a rationale in that process. The maturation of the human microbiota may be an example of ecological succession, in which communities undergo consecutive compositional and functional changes following initial colonization until a relatively stable “climax community” is established [43]. Hence, we provide the first evidence that neonates who will develop FI, during the first days of life, may have a peculiar gut microbiota environment characterized by an increased evenness, not allowing for a healthy gut colonization.

This hypothesis is also supported by evidence regarding the protective effects of breast milk versus NEC. Infants who are fed cow’s milk formula harbor more bacteria, showing a higher alpha diversity and more mature microbial pattern, as compared with exclusively breastfed infants [44].

The development of the microbiome follows a deterministic transition. In addition to microbial succession, the neonatal immune system also develops gradually in this critical period, exhibiting co-development between the microbiome and immune system [44]. Additionally, as has been described in the occurrence of gastro-intestinal tumors, the intestinal altered microbiota, characterized by increased evenness, might affect gastro-intestinal immune system maturation, and, thus, it could negatively influence the occurrence and development of FI, in preterm neonates, through its products, immune pathways and toxin secretion pathways [35].

Despite being original, the results of our study should be interpreted considering some limitations. The association between alpha-diversity evenness at birth and FI may be related to the effects of chance (random error), bias or confounding factors. In order to limit the risk of bias, we corrected our results for confounding variables. In addition, we reduced selection and spectrum biases by adopting strict inclusion and exclusion criteria and giving physicians proper information about the study methodology through several meetings. Moreover, we considered all the eligible patients and enrolled both intolerant and tolerant neonates consecutively. In order to minimize information bias, clinical data were collected by researchers different from those who performed metagenomic DNA extraction and sequencing of fecal samples. Researchers who collected clinical data were unaware of the results of microbiota analyses. As the diagnosis of FI is mainly based on clinical signs, the risk of misclassification bias was high. In order to improve this aspect, the classification of enrolled newborns was confirmed after an agreement between researchers. Analysis of intestinal microflora was performed by a researcher who was blinded regarding clinical information. Statistical analysis was performed by a statistician who was unaware of the study aims. The limited number of newborns reduces the generalizability of the results. The small sample size is related, at least in part, to the inclusion criteria adopted in this study and to the difficulty in fecal sampling within the first days of life. Further studies, specially designed to evaluate the relationship between Pielou evenness at birth and FI, are advocated.

## 5. Conclusions

Our results provide new insights about the association between gastro-intestinal microbiota diversity, in terms of evenness, and the identification of preterm neonates with a high level of immaturity in the gastro-intestinal tract, leading to intolerance for enteral nutrition. We support the hypothesis that the lack of establishment of pioneering bacterial colonia overcoming other minor taxa may not enable the microbial priming that helps seed the infant gut microbiota. These observations provide solid bases that may help to identify, in order to translate, new strategies improving FI through drug manipulation of preterm gut microflora. In particular, further trials should focus on the identification of nutritional and therapeutic strategies that foster the healthy microbial colonization of the gut, on the one hand promoting pioneer bacteria and, on the other hand, by preventing the colonization of those bacteria behaving as *Proteobacteria* in the early life of preterm neonates.

## Figures and Tables

**Table 1:** Baseline clinical characteristics of neonates included in the study.

	Intolerant *n* = 14	Tolerant *n* = 13	*p*
**Prenatal characteristics**			
Antenatal corticosteroids, No. (%)	11 (84.6)	10 (76.9)	0.500
IUGR, No. (%)	4 (28.6)	3 (23.1)	0.546
Pregnancy-induced hypertension, No. (%)	4 (28.6)	4 (30.8)	0.615
Hypothyroidism, No. (%)	4 (30.8)	2 (15.4)	0.322
Twins, No. (%)	8 (61.5)	3 (23.1)	0.055
Gestational Diabetes, No. (%)	1 (7.1)	3 (23.1)	0.269
Mother’s age ≥ 35 years old, No. (%)	7 (63.6)	6 (46.2)	0.329
Cesarean section, No. (%)	14 (100)	12 (92.3)	0.500
**Perinatal characteristics**			
Gestational age, weeks	30.2 (28.9 to 31.4)	29.2 (28.0 to 30.3)	0.219
Birth weight, g	1281.7 (1139.7 to 1423.8)	1280.2 (1044.2 to 1516.1)	0.990
5-min Apgar score	7.86 (7.36 to 8.36)	8.46 (7.83 to 9.10)	0.114
pH at birth	7.25 (7.21 to 7.28)	7.24 (7.15 to 7.32)	0.809
Base excess on cord blood, mmol/L	−7.09 (−9.96 to −4.22)	−6.27 (−9.78 to −2.76)	0.699
CRIB II score	4.75 (2.00 to 7.50)	6.83 (5.23 to 8.43)	0.125
Breast milk, g/kg/1st week	88.0 (27.4 to 148.7)	84.7 (13.5 to 155.9)	0.938

**Table 2:** Binary logistic regression to evaluate the influence of variables on the occurrence of FI.

Variables	ß	S.E.	Wald	*p* Value	Odds Ratio (OR)	95 C.I. for OR	
						Lower	Upper
VLBW	0.348	1.217	0.082	0.775	1.416	0.130	15.392
MEF	0.128	1.103	0.014	0.907	1.137	0.131	9.879
pH on cord blood ^°^	0.515	1.310	0.154	0.694	1.673	0.128	21.79
Pielou-evenness ^±^	2.486	1.035	5.769	0.016 *	12.018	1.580	91.40

VLBW = birth weight ≤1500 g; MEF = minimal enteral feeding. ^°^ pH on cord blood ≤ 7.2; ^±^ Pielou-evenness ≤ 0.5. * *p* < 0.05.

## Data Availability

The datasets analyzed during the current study are available from the corresponding author on reasonable request.
